# Brain Cholesterol Biosynthetic Pathway Is Altered in a Preclinical Model of Fragile X Syndrome

**DOI:** 10.3390/ijms23063408

**Published:** 2022-03-21

**Authors:** Martina Parente, Claudia Tonini, Valeria Buzzelli, Emilia Carbone, Viviana Trezza, Valentina Pallottini

**Affiliations:** 1 Department of Science, Roma Tre University, Viale Marconi 446, 00146 Rome, Italy; martina.parente@uniroma3.it (M.P.); claudia.tonini@uniroma3.it (C.T.); valeria.buzzelli@uniroma3.it (V.B.); emilia.carbone@uniroma3.it (E.C.); viviana.trezza@uniroma3.it (V.T.); 2 Neuroendocrinology Metabolism and Neuropharmacology Unit, IRCSS Fondazione Santa Lucia, Via del Fosso Fiorano 64, 00143 Rome, Italy

**Keywords:** 3-Hydroxy 3-methylglutaryl Coenzyme A reductase, brain, cholesterol, *Fmr1-Δexon 8* rat, Fragile X Syndrome, low-density lipoprotein receptor, liver, plasma, prenylated proteins

## Abstract

Fragile X Syndrome (FXS) is the most frequent form of inherited X-linked pathology, associated with an intellectual and developmental disability, and currently considered the first monogenic cause of autism spectrum disorder (ASD). Low levels of total cholesterol reported in the serum of FXS patients, and evidence that FMRP targets a subset of mRNAs encoding proteins of lipid synthesis and transport suggests that the cholesterol metabolism impairments could be involved in FXS. Thus, the aim of the presented work was to investigate the modulations of the cholesterol biosynthetic pathway and its end-products in a recently developed *Fmr1-Δexon 8* rat model of FXS. Here, we show that this experimental model mimics what is found in FXS patients, exhibiting a lower serum cholesterol content, accompanied by a reduction in food intake and body weight compared to WT animals. Moreover, alterations of proteins committed to cholesterol synthesis and uptake have been observed in the amygdala, prefrontal cortex and nucleus accumbens. Interestingly, the end-products show a brain region-dependent modulation in *Fmr1-Δexon 8* rats. Overall, our results demonstrate that the cholesterol biosynthetic pathway is altered in some brain regions of this preclinical model of FXS. This finding has relevance for future studies to delve deeper into the involvement of this metabolic process in FXS, and thus its possible role as a therapeutic target.

## 1. Introduction

**Figure 1:**
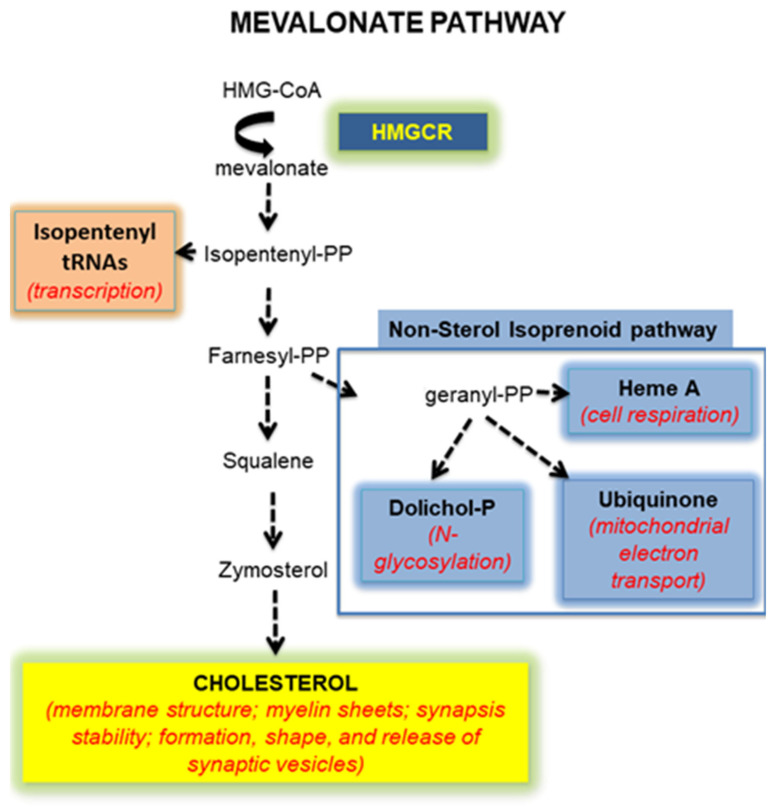
A schematic illustration of the mevalonate pathway.

Autism spectrum disorder (ASD) is described as a group of neurodevelopmental diseases, the symptoms of which begin during early childhood and typically last throughout a person’s life. The causes and risk factors that make children more likely to develop ASD are manifold, including environmental, biological, and genetic influences [1]. Fragile X Syndrome (FXS) is the most frequent form of inherited X-linked pathology and the first monogenic cause of ASD. FXS shows a prevalence of 1:4000 males and 1:7000 females [2] who present severe intellectual disability accompanied by deficits in learning and memory. FXS is caused by the abnormal expansion of the triplet CCG in the 5′-UTR region of the *Fmr1* gene, which leads to its hypermethylation and silencing, and a consequent strong reduction or even loss of gene product expression: the Fragile X mental retardation protein (FMRP) [3,4]. FMRP is an RNA-binding protein regulating mRNA translation, trafficking and stability [5]. FMRP is widely expressed in the brain, where it binds a great number of mRNAs encoding for proteins with an important role for neuronal function and maturation, and synaptic plasticity [6,7]. In fact, the critical reduction or the absence of FMRP l, causes the failure in the synapse maturation and dendrite elongation observed in FXS patients and in *Fmr1* knock-out animal models [8]. It has been demonstrated that dendrite initiation, elongation and branching are stimulated by the Rho GTPase family, the best studied of which are Rac1, RhoA and Cdc42 [9,10]. In addition, RhoA has been related to the activation of the cAMP response element-binding protein (CREB) which is a transcription factor involved in long-term memory [11,12] and in several brain functions [13], including the fundamental neurotrophin production [14,15]. All these small GTPase need post-translational modification to target them to cellular membranes and be active [16,17]. Specifically, these signaling proteins undergo prenylation which consists of a covalent attachment of farnesyl or geranylgeranyl, two types of prenyls produced by the non-sterol isoprenoid pathway, a branch of the mevalonate (MVA) pathway which is the biochemical process synthesizing cholesterol (Figure 1) [18].

The MVA pathway consists of a long sequence of enzymatic steps whose rate-limiting enzyme is the 3-Hydroxy 3-methylglutaryl coenzyme A reductase (HMGCR) [19]. Besides cholesterol and prenyls, several other molecules (isopentenyl tRNAs, dolichol phosphate and ubiquinone) are crucial for numerous cellular processes derived from the MVA pathway (Figure 1) [20]. The importance of its end-products underlines the key physiological role of the MVA pathway, and how it must be precisely regulated to avoid cell dysfunctions and in turn pathological implications [21]. As an example of its involvement in a neurodevelopmental disease, the MVA pathway results altered in dependence of the region, the sex and the age in the brain of a preclinical model of ASD, such as rats prenatally exposed to valproate [22,23,24]. Very interestingly, it has been demonstrated that the pharmacological inhibition of the MVA pathway corrects the excessive hippocampal protein synthesis and susceptibility to audiogenic seizures displayed by FXS mice, suggesting a potential role of the cholesterol biosynthetic pathway in this pathology [25]. Moreover, statistically significant lower levels of total cholesterol and HDL-cholesterol have been observed in the serum of FXS patients if compared to the normal population [26,27]. Additionally, it was found that FMRP targets a subset of mRNA encoding proteins critically involved in lipid synthesis and transport [6]. Together, these observations support the hypothesis that the MVA pathway could be modulated in FXS, but data focused on the putative alteration of this complex physiological process are lacking. Thus, the presented work was aimed at investigating the MVA pathway and its end-products in plasma, liver, and in six different brain regions of a recently developed *Fmr1-Δexon 8* rat model of FXS [28,29,30]. This preclinical model has been proposed as the first rat model of FXS.

In this model, the inactivation of the *FMR1* gene was performed using zinc-finger nucleases technology (ZFN) [31,32]. Recently, it has been shown that this model is not a null KO of *Fmr1*, but instead, the induced mutation results in a gene product with a loss of exon 8, which encodes a domain responsible for RNA-binding, named KH1 domain [33]. Such deletion is sufficient to cause FXS-like traits, such as deficits in sustained visuospatial attention [34], altered patterns of social interaction, and cognitive impairment [28], together with abnormal synaptic plasticity [32,35,36]. The fact that rats are easier to train, can learn sophisticated behaviors and have a more elaborated social repertoire than mice, make the *Fmr1-Δexon 8* rat a valuable tool to study the neurobiological aspects of FXS.

## 2. Results

In the *Fmr1-^Δ^exon 8* rat model, the amount of serum cholesterol was measured to uncover if this FXS model shares some metabolic features observable in human beings affected by this pathology. Our results showed a decreased serum cholesterol content in both PND35 (adolescent) and PND90 (adult) *Fmr1-^Δ^exon 8* rats, compared to WT (Table 1).

**Figure 2:**
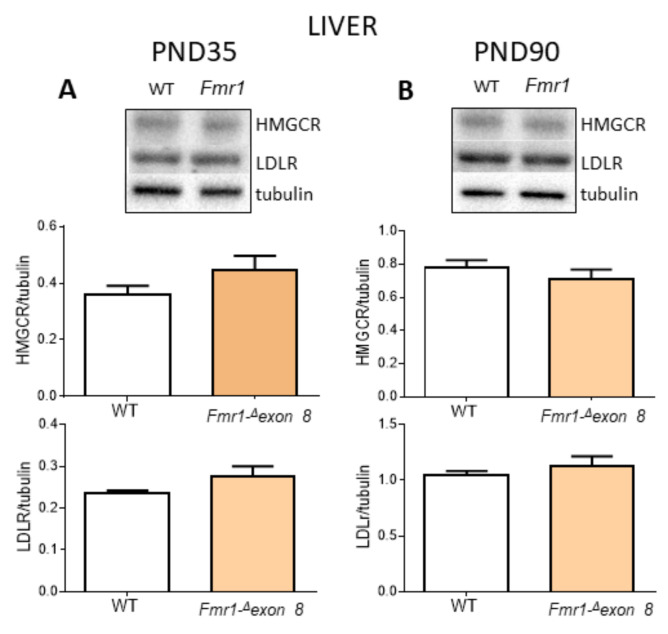
HMGCR and LDLR protein level in the liver of *Fmr1-^Δ^exon 8* and WT male adolescent (PND35) and adult (PND90) rats. (**A**) illustrates a typical Western blot (top) and the densitometric analysis of HMGCR and LDLR in PND35 rats. (**B**) illustrates a typical Western blot (top) and the densitometric analysis of HMGCR and LDLR in PND90 rats. Values represent mean ± SD obtained from 6 animals performed in duplicate. Tubulin served as a housekeeping protein to normalize protein loading. Statistical analysis was carried out by using the unpaired Student’s *t*-test.

To understand the cause of the reduction in the serum cholesterol content, we investigated the cholesterol metabolism in the liver, the metabolic power station of lipids [37]. Specifically, we analyzed the protein levels of HMGCR and the low-density lipoprotein receptor (LDLR) which are committed to cholesterol synthesis and uptake, respectively [19]. No changes in both proteins were observed either during adolescence (Figure 2A) or adult age (Figure 2B), and no changes in hepatic cholesterol content between *Fmr1-Δexon 8* rats and WT animals were detected (Table 2).

**Figure 3:**
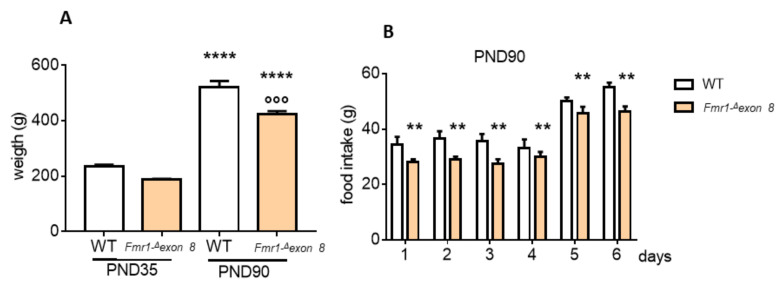
Body weight and food intake measurements of the *Fmr1-^Δ^exon 8* and WT male rats. (**A**) shows the weight of the *Fmr1-^Δ^exon 8* and WT adolescent (PND35) and adult (PND90) rats at the moment of euthanasia. (**B**) illustrates the grams of food intake of *Fmr1-^Δ^exon 8* and WT PND90 rats, measured during the last 6 days before euthanasia. Values represent mean ± SD obtained from 6 animals performed in duplicate. Statistical analysis was carried out by using the unpaired Student’s *t*-test. °°° = *p* < 0.001 vs. WT PND90; **** = *p* < *0*.00001 vs. PND35; ** = *p* < 0.01 vs. WT.

We postulated that the lower cholesterolemia occurring in *Fmr1-^Δ^exon 8* rats could depend on a lower food intake. Indeed, we observed a reduction in body weight of *Fmr1-^Δ^exon 8* rats, with adults weighing about 18% less than their WT controls. Figure 3B shows that food intake was reduced in *Fmr1-^Δ^exon 8* adult rats than WT animals, supporting our hypothesis.

**Figure 4:**
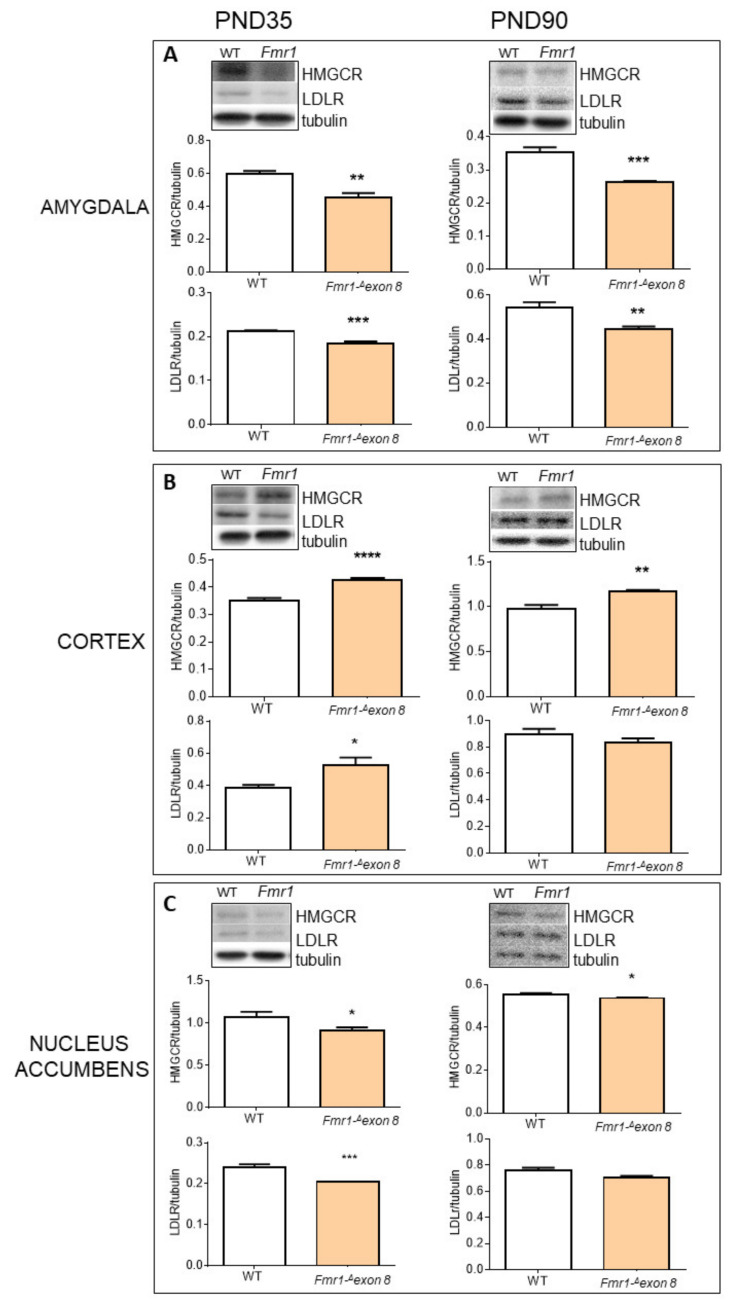
HMGCR and LDLR protein level in brain areas of *Fmr1-^Δ^exon 8* and WT male adolescent (PND35) and adult (PND90) rats. (**A**) illustrates a typical Western blot (top) and the densitometric analysis of HMGCR and LDLR in the amygdala of PND35 and PND90 rats. (**B**) illustrates a typical Western blot (top) and the densitometric analyses of HMGCR and LDLR in the prefrontal cortex (CORTEX) of PND35 and PND90 rats. (**C**) illustrates a typical Western blot (top) and the densitometric analyses of HMGCR and LDLR in nucleus accumbens of PND35 and PND90 rats. Tubulin served as a housekeeping protein to normalize protein loading. Values represent mean ± SD obtained from 6 animals performed in duplicate. Statistical analysis was carried out by using the unpaired Student’s *t*-test. * = *p* < 0.05 vs. WT; ** = *p* < 0.01 vs. WT; *** = *p* < 0.001 vs. WT; **** = *p* < 0.0001 vs. WT.

Successively, we focused our attention on selected brain areas (amygdala, cerebellum, cortex, hippocampus, nucleus accumbens and dorsal striatum) known to be involved in ASD [22,38,39]. As shown in Figure 4, HMGCR and LDLR were altered in the brain of *Fmr1-Δexon 8*. In particular, in the amygdala (Figure 4A) and nucleus accumbens (Figure 4C) both HMGCR and LDLR decreased in *Fmr1-Δexon 8* rats compared to WT animals, either in PND35 and PND90 rats. On the contrary, in the prefrontal cortex (Figure 4B) HMGCR and LDLR were higher in *Fmr1-Δexon 8* than WT controls, at both ages under investigation. Finally, in the cerebellum, hippocampus and dorsal striatum, no changes were observed in *Fmr1-Δexon 8* animals (Supplementary Figure S1).

On the basis of these results, we investigated the end-products of the MVA pathway likely related to the modulation of HMGCR. Cholesterol is the main end-product, whose production is tightly controlled in the brain. In this second part, we concentrated on PND90 animals to assess the effects of persistent changes in HMGCR. Checking the cholesterol content in the amygdala, prefrontal cortex and nucleus accumbens—the cerebral regions showing an HMGCR alteration,—we found that tissue cholesterol content was altered only in the amygdala (Table 3), while in the prefrontal cortex and nucleus accumbens, the modulations of HMGCR were not accompanied by changes in cholesterol tissue content.

**Figure 5:**
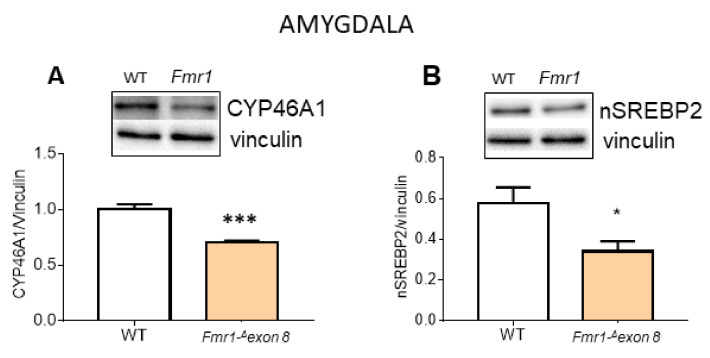
CYP46A1 and nSREBP2 protein level in amygdala of *Fmr1-^Δ^exon 8* and WT adult (PND90) male rats. (**A**) illustrates a typical Western blot (top) and the densitometric analyses of CYP46A1 in the amygdala of PND90 rats. (**B**) illustrates a typical Western blot (top) and the densitometric analyses of nSREBP2 in the amygdala of PND90 rats. Vinculin served as a housekeeping protein to normalize protein loading. Values represent mean ± SD obtained from 6 animals performed in duplicate. Statistical analysis was carried out by using the unpaired Student’s *t*-test. * = *p* < 0.05 vs. WT; *** = *p* < 0.001 vs. WT.

The increased cholesterol tissue content was not explained by a coherent decline of cholesterol synthesis or uptake in the amygdala. To identify a possible cause, cytochrome P450 46A1 (CYP46A1) protein was measured. CYP46A1 plays a major role in cholesterol homeostasis in the brain since it catalyzes the hydroxylation at C-24 of the cholesterol side chain, thus triggering cholesterol diffusion out of neurons and its further degradation in the liver [40]. As hypothesized, CYP46A1 decreased in *Fmr1-Δexon 8* rats compared to WT animals (Figure 5A). These results may suggest that the CYP46A1-dependent accumulation of cholesterol could mediate the inhibition of synthesis and import processes by a classical homeostatic response [19]. Of course, further investigations are needed, however, our hypothesis is supported by the reduction in a nuclear and transcriptional active fragment of the sterol regulatory binding protein 2 (nSREBP2), committed to all the genes involved in cholesterol metabolism, which we detected in the amygdala of *Fmr1-Δexon 8* rats (Figure 5B).

**Figure 6:**
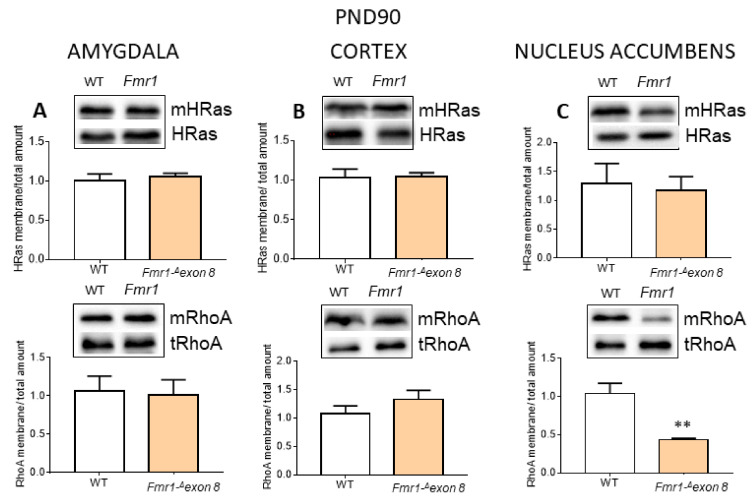
Membrane-bound and total level ratio of HRas and RhoA protein content in brain areas of *Fmr1-^Δ^exon 8* and WT adult (PND90) male rats. The figure illustrates a typical Western blot and a graph representing the ratio of the densitometric analysis between membrane-bound and total levels of HRas (top) and RhoA (bottom) in PND90 rats. The loading control was performed by using caveolin 1 (Figure S3). (**A**): amygdala; (**B**): prefrontal cortex (cortex); (**C**): nucleus accumbens. Values represent mean ± SD obtained from 6 animals performed in duplicate. Statistical analysis was carried out by using the unpaired Student’s *t*-test. ** = *p* < 0.01 vs. WT.

Additional end-products of the cholesterol biosynthetic pathway are represented by prenyls, farnesyl and geranylgeranyl, which are covalently bound to small GTPase inducing their membrane translocation and activation [41]. We studied two small GTPase, HRAS and RhoA, as prototypes of farnesylation and geranylgeranylation, respectively. The data were obtained by performing the ratio between the membrane-bound protein and the cellular total content, which provides the cellular amount of the prenylated proteins. We showed that the activated RhoA levels decreased in *Fmr1-^Δ^exon 8* rats compared to WT animals, in agreement with the modulation of HMGCR only in the nucleus accumbens (Figure 6C).

## 3. Discussion

ASD is a complex and high heterogeneous neurodevelopmental disease with multiple etiologies, sub-types, and clinical presentation. FXS is the most frequent form of inherited X-linked pathology, and, the first monogenic cause of ASD. Thus far, no treatments for this pathology exist, and researchers sought to determine the neurobiological alterations and to find prospective therapeutic targets. Here, we used the recently developed *Fmr1-Δexon 8* rat model of FXS [28] to study the involvement of the cholesterol biosynthetic pathway, the so-called MVA pathway, in FXS. It is widely supported that cholesterol metabolism is altered in numerous neurological pathologies, including ASD [22]. Our study demonstrates that the *Fmr1-Δexon 8* preclinical model mimics the reduced plasma cholesterol content observed in FXS patients [26,27]. Unexpectedly, the hepatic key proteins which maintain cholesterol homeostasis (HMGCR and LDLR) seem not involved, although 80% of the cholesterol present in the mammal body is endogenously synthesized, mainly in the liver [42]. Coherently with these results, tissue cholesterol content was unchanged in *Fmr1-Δexon 8* animals. Considering the slightly reduced animal body weight of *Fmr1-Δexon 8* rats, their decreased cholesterolemia could depend on a reduced food intake. Effectively, the daily measurement of food intake showed that *Fmr1-Δexon 8* animals eat less than their WT counterparts. Our results are supported by previous work conducted by Long and collaborators [43]. Indeed, they have recently demonstrated that X fragile protein-deficient mice present decreased agouti-related peptide (AgRP), which is one of the most potent and long-lasting appetite stimulators [44]. Together, the reduced appetite stimulation and the consequent reduction in food intake and body weight may be at the root of the lower cholesterolemia observable in *Fmr1-Δexon 8* rats. Of course, we cannot exclude that other metabolic mechanisms can contribute to the plasma cholesterol imbalance observed in this FXS model.

Regarding the brain, it is important to note that in this organ, cholesterol metabolism is almost completely separated from the rest of the body because the blood-brain barrier (BBB) prevents the passage of peripheric lipoproteins [45]. Consequently, all the cholesterol present in the brain is synthesized in situ, and its content and metabolism do not depend on either hepatic production or diet intake. Interestingly, we observed alterations in three out of the six brain regions that were investigated. In particular, the amygdala, nucleus accumbens and prefrontal cortex showed altered levels of HMGCR and LDLR expression, while in the cerebellum, hippocampus and dorsal striatum, these proteins seemed to be not affected by FMR1 protein deficiency. As also previously observed [22,46], these data indicate different cholesterol metabolism modulations related to some brain regions in ASD. The involvement of cholesterol homeostasis dysfunctions in FXS is further supported by the evidence that HMGCR inhibitors partially correct symptoms in Fmr1 knock-out mice [47], suggesting a putative role of the MVA pathway in FXS manifestations, at least in animal models. Reasonably, alterations in HMGCR and LDLR levels change cholesterol synthesis and uptake rate, which may be followed by changes in tissue cholesterol content. Among the brain areas analyzed, surprisingly, we observed an accumulation of cholesterol only in the amygdala of *Fmr1-Δexon 8* animals where HMGCR decreases. We speculate that the increased cholesterol content could be induced by the CYP46A1 reduction observed in these animals. Indeed, CYP46A1 is a key brain-specific enzyme that converts cholesterol to 24-hydroxycholesterol (24 HC), which is able to cross the BBB and to reach the liver to be degraded [48]. Interestingly, modulation of CYP46A1 has been indirectly demonstrated in autistic children, who showed higher plasma 24 HC levels, which progressively decreased with age [49]. Thus, the CYP46A1-dependent cholesterol accumulation in the amygdala may induce the classic homeostatic response which suppresses HMGCR and LDLR protein expression through nSREBP2 reduction [19]. Intriguingly, an altered brain cholesterol content has been related not only to ASD [22,46,50] but also to several neurodegenerative diseases [51] and psychiatric diseases [23]. Another important issue is the non-sterol branch of the MVA pathway responsible for isoprenoid synthesis, and it is ultimately essential in a plethora of neurological functions [52]. In the amygdala, we did not observe any change in the prenylated proteins that we measured; therefore, in this area, it seems that cholesterol biosynthetic pathway alteration in *Fmr1-Δexon 8* rats is driven by the unbalanced rate of cholesterol degradation despite a direct effect on HMGCR. Regarding the prefrontal cortex, the increase in HMGCR and LDLR is slightly stronger in the adolescent *Fmr1-Δexon 8* rats than in adult animals, which showed an LDLR protein content similar to adult WT animals, and the increase in HMGCR protein displays a less robust statistical significance. Moreover, no changes in the end-products of cholesterol biosynthetic pathway have been observed. This suggests that in our FXS model, cholesterol metabolism in the prefrontal cortex is less affected by FMR1 protein deficiency. Notably, in line with this observation, our previous work, carried out on rats with valproic acid (VPA)-induced autistic phenotype, showed that no alterations of HMGCR occurred in the prefrontal cortex during both adolescence and adult age, while an increase in LDLR was observable only in adult rats [22]. Obviously, a precise overlap between the two different experimental models cannot occur, as they are based on genetic (*Fmr1* gene manipulation) and environmental (prenatal VPA exposure) factors, differently involved in syndromic and non-syndromic forms of ASD. Concerning the nucleus accumbens, the reduction in HMGCR levels is accompanied by a decrease in RhoA activation, whereas no cholesterol alteration was detected in the tissue. RhoA has been involved in key neurobiological processes. It integrates molecular signals to organize sophisticated and coordinated variations in gene expression and actin cytoskeleton, crucial processes for the neurite outgrowth and synaptic connectivity [53,54]. Therefore, it is not surprising that RhoA activity has been related to developmental disabilities, such as mental retardation [55]. For instance, we have previously demonstrated that HMGCR inhibition, and in turn the reduction in RhoA activation, are deeply involved in neurite outgrowth [10]. Furthermore, it has been shown that drugs interfering with this axis impacts neuronal morphology and function [56,57,58].

Taken together, our results demonstrate for the first time that the cholesterol biosynthetic pathway is altered in some brain regions of a preclinical model of FXS. The cholesterol plasma alterations observable in patients [26,27] are excellently mimicked by *Fmr1-Δexon 8* rats, making this experimental model very useful in this research field. The modulations in cholesterol metabolism observed in each brain area need to be clarified by further studies, trying to relate them to different variations in functioning, and moreover, it would be very interesting to study the specific cellular types involved. In addition, we cannot exclude that other lipids could be modulated in our experimental model. Indeed, some receptors activated by lipids (liver X receptor, retinoid X receptor, farnesoid X receptor), as well as several apolipoproteins (APOA1, APOA2, APOA4, APOC2 and APOD) are modulated in the cerebrospinal fluid of Fragile X-associated tremor/ataxia syndrome patients, as recently demonstrated [59]. Moreover, we did not consider the potential differences between hemizygous males and heterozygous females; this is a very interesting point since cholesterol metabolism displays sex-dependent differences [24], and this issue deserves further evaluation.

In conclusion, our findings, together with previous results obtained in environmentally-triggered animal models of ASD [22,46], reinforce the presence of brain changes in cholesterol metabolism, thus, setting the basis for future studies to delve deeper into the involvement of cholesterol biosynthetic pathways in ASD and its possible role as a therapeutic target.

## 4. Materials and Methods

### 4.1. Animals and Sample Collection

In the FXS model used in this work, and named *Fmr1-^Δ^exon 8 rats*, the absence of the protein product is given by a mutation that is induced in the exon 8 of the *FMR1* gene through zinc-finger nucleases (ZFN) technology in the outbred Sprague-Dawley background [32]. The design and cloning of the ZFN, as well as the embryonic microinjection and screening for positive founder rats, were performed by SAGE Labs (Boyertown, PA, USA), as previously described [60].

Pregnant *Fmr1-Δexon 8* rats and the corresponding wild-type (WT) controls were individually housed in Makrolon cages (40 (l) × 26 (w) × 20 (h) cm), under controlled conditions (temperature 20–21 °C, 55–65% relative humidity and a 12/12 h light cycle with lights on at 07:00 h). Newborn litters found up to 17:00 h were considered to be born on that day (postnatal day (PND 0). On PND 1, the litters were culled to eight animals (six males and two females) to reduce any litter size-induced variability in the growth and development of pups during the postnatal period. On PND 21, the pups were weaned and housed in groups of three (of the same sex and same genotype) till adulthood. For the experiments, only males were used, since FXS is an X-linked disease, thus females were discarded. During the last 6 days of life of the PND90 rats, food intake was measured weighing the food of each cage every day at 10:00 o’clock, and the cumulative food intake was calculated by subtracting the weight of the residual food from the one weighed the day before. At PND35 or PND90, rats were rapidly decapitated, blood was collected, livers and brains were removed. Blood was clotted at RT for 15 min, then centrifuged at 5000× *g*, and the serum was transferred to 1 mL tubes. The amygdala, cerebellum, prefrontal cortex (cortex), hippocampus, nucleus accumbens and dorsal striatum were dissected by hand under microscopic control within 2 min.

The experiments were performed in agreement with the ARRIVE (Animals in Research: Reporting In Vivo Experiments) guidelines, the guidelines of the Italian Ministry of Health (D.L. 26/14), and the European Community Directive 2010/63/EU. Italian Ministry of Health authorization 608/2015-PR.

### 4.2. Measurement of Cholesterol in Brain, Liver and Serum Samples

The cholesterol amount in tissues and serum were measured by using the Cholesterol Quantitation Kit-MAK043 (Sigma-Aldrich, Milan, Italy), following the manufacturer’s instructions. Briefly, 10 mg of hepatic tissues and of each brain area were lysed in 200 µL of chloroform:isopropanol:nonylphenylpolyethylene glycol solution (Nonidet P-40) (7:11:0.1). The extracted lipids were resuspended in buffer solution and 5 µL were used for each sample. For the serum cholesterol measurement, 2 µL of the sample were directly added to the reaction mix. The assay detects total cholesterol (cholesterol and cholesteryl esters) when cholesterol esterase is included in the reaction, or free cholesterol when it is not included. The amount of cholesteryl ester can be determined by subtracting the value of free cholesterol from the total (cholesterol plus cholesteryl esters). The enzymatic assay results in a colorimetric product, proportional to the cholesterol present in the sample. The amount of cholesterol present in the samples was revealed by determining the absorbance at 570 nm with the Tecan Spark microplate reader (Männedorf, Switzerland). All samples were run in duplicate.

### 4.3. Total Lysate and Membranes Preparation for Western Blot Analysis

Liver and brain homogenates were obtained by sonication (VCX 130 PB, Sonics, Newtown, 06470 CT, USA) of tissues in Sucrose 0.1 M, KCl 0.05 M, KH2PO4 0.04 M, EDTA 0.04 M, pH 7.4 plus a 1:1000 protease inhibitor cocktail, and 1:400 phosphatase inhibitor cocktail (Sigma-Aldrich). Liver and brain samples were prepared at 1:10 and 1:5 *w*/*v*, respectively, to yield total lysate. Membrane fractions were isolated by centrifuging the total lysate at 22,000× *g* for 1 h at 4 °C, and then solubilizing the pellet in homogenization buffer by sonication. Purity control of the obtained membrane fraction was performed by checking the Western blot tubulin content, a purely cytosolic protein (Supplementary Figure S2). Total proteins were quantified by the method of Lowry [61].

### 4.4. Immunoblotting

Solubilized proteins (30 µg) from total lysates or isolated membrane fractions were diluted with Laemmli buffer, boiled for 5 min and resolved by 7% or 13.5% SDS-PAGE at 50 mA first, and then at 120 V. Subsequently, proteins were transferred to nitrocellulose membrane using the Trans-Blot Turbo Transfer System (Bio-Rad Laboratories) for 10 min at 25 V. The membranes were blocked with 5% fat-free milk in Tris-buffered saline (NaCl 0.138 M, KCl 0.027 M, Tris-HCl 0.025 M, and 0.05% Tween-20, pH 6.8) for 1 h at RT, and then incubated overnight at 4 °C, with primary antibody. Finally, incubation for 1 h at RT with secondary peroxidase-conjugated antibody produced in mouse or in rabbit (1:10,000; Bio-Rad) was performed to reveal the immunoreactivity. Bands were detected using a ChemiDocTM apparatus (Bio-Rad Laboratories, Milan, Italy). Western blot images were analyzed by ImageJ (National Institutes of Health, Bethesda, MD, USA) software for Windows. Intensities of proteins of interest were normalized to intensities of respective housekeeping proteins. The following primary antibodies were tested: HMGCR (Abcam, ab242315, dilution 1:1000), LDLR (Abcam, ab30532, dilution 1:1000), RhoA (Santa Cruz Biotechnology, Sanya Cruz, CA, USA, sc-418, dilution 1:500), HRas (Santa Cruz Biotechnology, sc-53959, dilution 1:500), CYP46A1 (Santa Cruz Biotechnology, sc-136148, dilution 1:1000), nuclear-SREBP2 (abcam, ab28482, dilution 1:1000). Antibodies against tubulin or vinculin (Sigma-Aldrich, Milan, Italy, dilution 1:10,000) were chosen as a loading control. Different housekeeping proteins were used depending on the molecular weight of the analyzed protein, in order to avoid confounding signals when detecting the immunoreactivity. Finally, caveolin 1 (Santa Cruz Biotechnology dilution 1:1000) was chosen as a loading control in experiments evaluating proteins in isolated membrane fractions.

### 4.5. Statistical Analysis

All data are represented as mean ± SD. The experiments were performed using six animals per experimental group. Statistically significant differences were tested by unpaired Student’s *t*-tests. Statistical analysis and graphical illustrations were performed with GraphPad Prism, version 8.0 (GraphPad, La Jolla, CA, USA) for Windows.

## Figures and Tables

**Table 1:** Serum cholesterol content in adolescent (PND35) and adult (PND90) male *Fmr1-^Δ^exon 8* rats and their WT counterpart.

Serum Cholesterol mg/dL			
Groups	WT	*Fmr1-^Δ^exon 8*	*p*
PND35	55.37 ± 7.98	44.53 ± 13.14 *	0.0233
PND90	73.38 ± 13.76	51.51 ± 4.55 **	0.0097

Cholesterol content is expressed as mg/dL serum. Values represent mean ± SD obtained from 6 animals performed in duplicate. Statistical analysis was performed by using the unpaired Student’s *t*-test. * = *p* < 0.5; ** = *p* < 0.01 vs. WT.

**Table 2:** Liver cholesterol content in male adolescent (PND35) and adult (PND90) *Fmr1-^Δ^exon 8* rats and their WT controls.

Liver Cholesterol Content mg/g Tissue			
Groups	WT	*Fmr1-^Δ^exon 8*	*p*
PND35	1.22 ± 0.28	1.08 ± 0.13	0.364
PND90	0.87 ± 0.10	1.04 ± 0.17	0.098

Cholesterol content is expressed as mg/g tissue. Values represent mean ± SD obtained from 6 animals performed in duplicate. Statistical analysis was performed by using the unpaired Student’s *t*-test.

**Table 3:** Cholesterol content in nucleus accumbens, amygdala and prefrontal cortex (cortex) of *Fmr1-^Δ^exon 8* and WT adult (PND90) male rats.

Brain Cholesterol Content mg/g Tissue			
Groups	WT	*Fmr1-^Δ^exon 8*	*p*
Nucleus accumbens	1.22 ± 0.28	1.08 ± 0.13	0.364
Cortex	6.02 ± 0.90	5.77 ± 0.80	0.644
Amygdala	5.30 ± 0.81	9.66 ± 1.97 **	0.004

Cholesterol content is expressed as mg/g tissue. Values represent mean ± SD obtained from 6 animals performed in duplicate. Statistical analyses were performed by using the unpaired Student’s *t*-test. ** = *p* < 0.01.

## Data Availability

The data presented in this study are available on request from the corresponding author.

## Supplementary Materials

The following supporting information can be downloaded at: https://www.mdpi.com/article/10.3390/ijms23063408/s1.
