# Supplementary material for: Brain Cholesterol Biosynthetic Pathway Is Altered in a Preclinical Model of Fragile X Syndrome
Source: Int J Mol Sci. 2022 Mar 21;23(6):3408. doi: 10.3390/ijms23063408 (PMC8955806; doi:10.3390/ijms23063408)

Supplementary 1

HMGCR and LDLR protein level in brain areas of Fmr1-Δexon 8 and WT male adolescent (PND35) and adult (PND90) rats

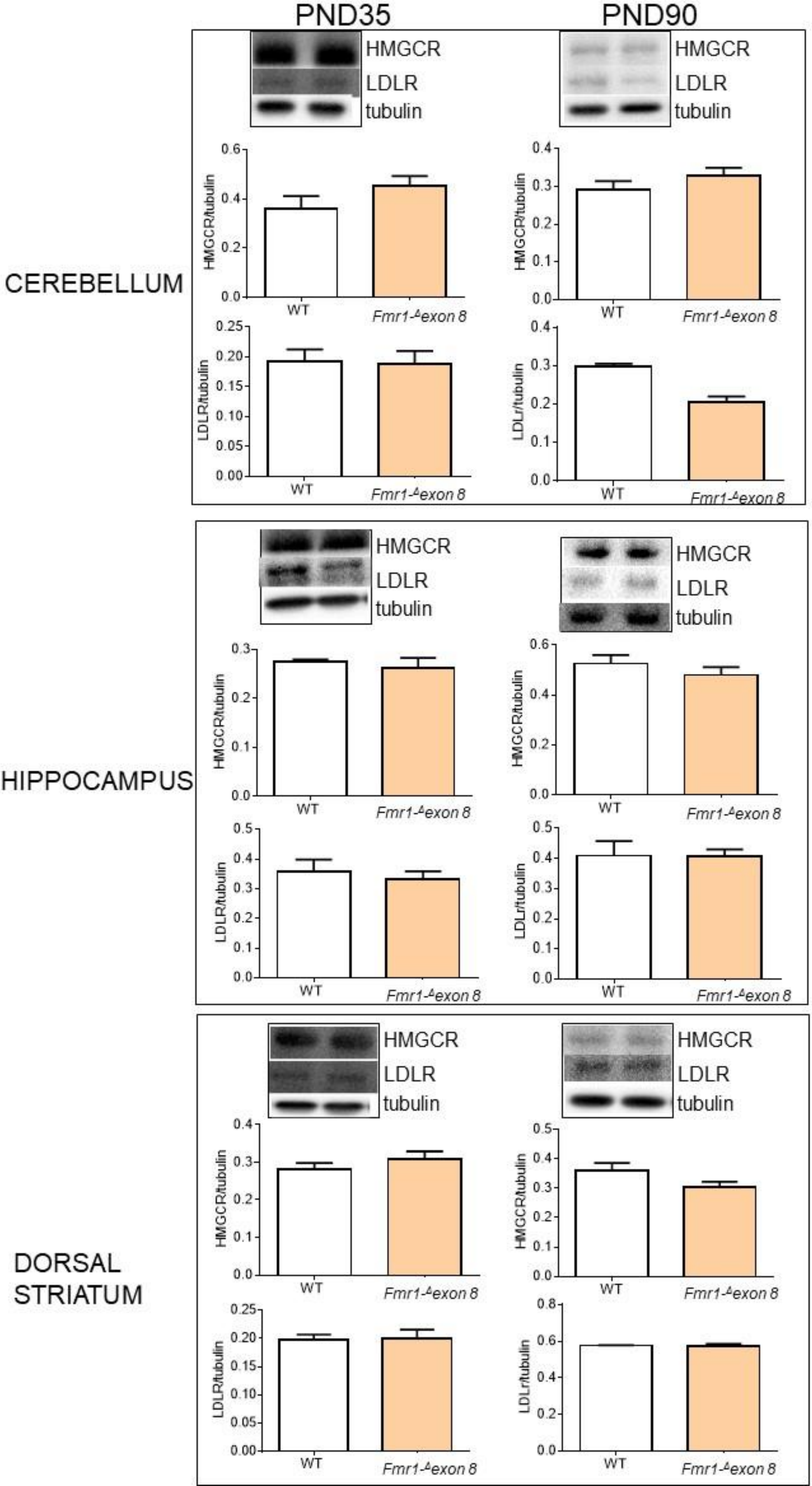

Figure S2

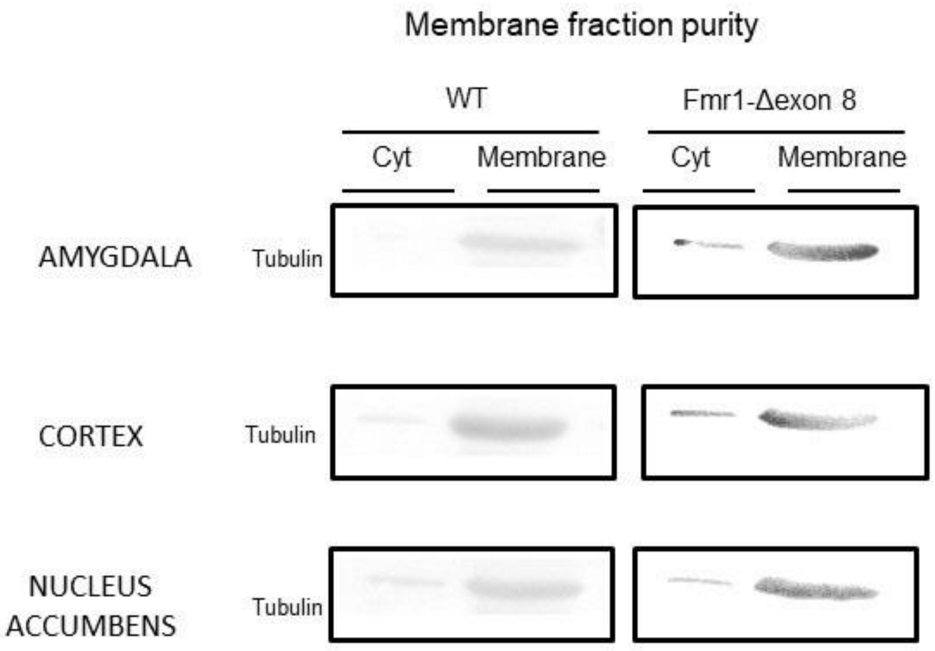

Membrane fraction purity
WT
Fmr1-Δexon 8
Cyt
Membrane
Cyt
Membrane
AMYGDALA
Tubulin
CORTEX
Tubulin
NUCLEUS ACCUMBENS
Tubulin

Figure S3

Membrane-bound and total level ratio of HRas and RhoA protein content in brain areas of Fmr1-Δexon 8 and WT adult (PND90) male rats

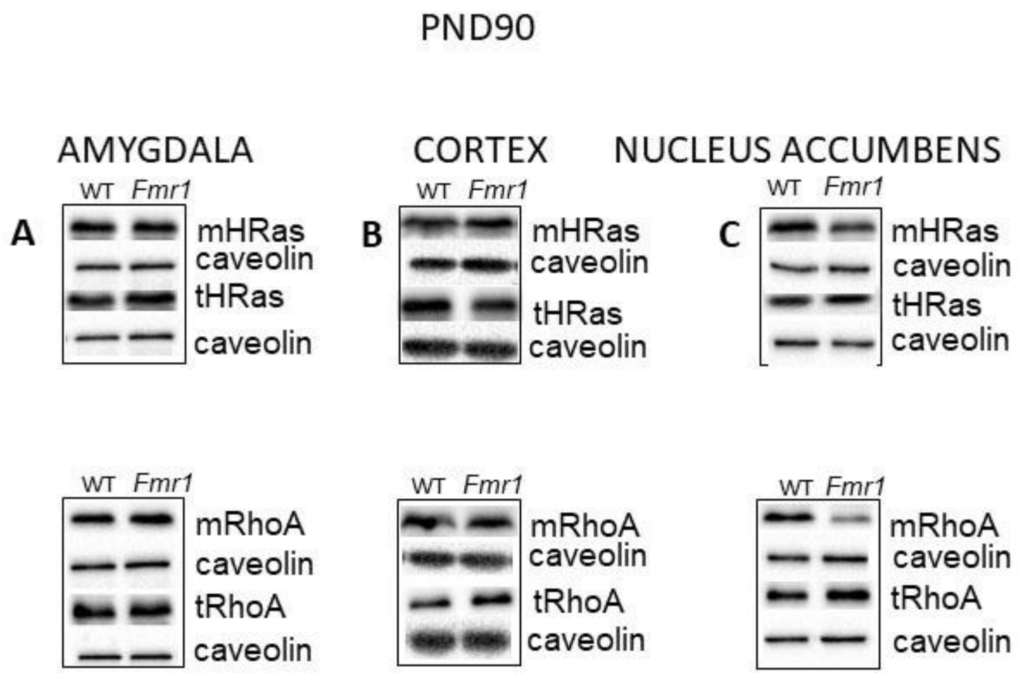

Supplement: Supplementary file 1 [file ijms-23-03408-s001.zip › ijms-1551439-supplementary.pdf]
